# Low-Dose, Long-Term *Callistemon citrinus* and Its Main Compounds Alter Hepatic Fatty Acid Profiles and Protect Liver Histology in Rats Fed a High-Fat–Sucrose Diet

**DOI:** 10.3390/ijms27167255

**Published:** 2026-08-14

**Authors:** Luis Alberto Ayala-Ruiz, Aram Josué García-Calderón, Oliver Rafid Magaña-Rodríguez, Adrián Sánchez-Orozco, Manuel López-Rodríguez, Joel E. López-Meza, Asdrubal Aguilera-Méndez, Patricia Rios-Chavez

**Affiliations:** 1 Facultad de Biología, Universidad Michoacana de San Nicolás de Hidalgo, Morelia 58030, Michoacán, Mexico; 1232816g@umich.mx (L.A.A.-R.); 1208935x@umich.mx (A.J.G.-C.); oliver.rodriguez@umich.mx (O.R.M.-R.); 2 Facultad de Medicina Veterinaria y Zootecnia, Universidad Michoacana de San Nicolás de Hidalgo, Tarímbaro 58893, Michoacán, Mexico; adrian.sanchez@umich.mx (A.S.-O.); manuel.lopez@umich.mx (M.L.-R.); 3 Centro Multidisciplinario de Estudios en Biotecnología, Facultad de Medicina Veterinaria y Zootecnia, Universidad Michoacana de San Nicolás de Hidalgo, Tarímbaro 58893, Michoacán, Mexico; elmeza@umich.mx; 4 Instituto de Investigaciones Químico Biológicas, Universidad Michoacana de San Nicolás de Hidalgo, Tarímbaro 58893, Michoacán, Mexico; amendez@umich.mx

**Keywords:** metabolic dysfunction–associated fatty liver disease, high-fat–sucrose diet

## Abstract

Diets high in fat and refined carbohydrates reliably mimic key features of metabolic dysfunction-associated fatty liver disease (MAFLD), including hepatic lipid accumulation, oxidative stress, and ongoing liver injury. This study evaluated the long-term, low-dose administration of *Callistemon citrinus* ethanolic leaf extract and its primary bioactive compounds: d-limonene, ellagic acid, gallic acid, and *p*-coumaric acid to prevent lipotoxicity in rats fed a high-fat–sucrose diet (HFSD). Sixty male Wistar rats were randomly divided into 10 groups: normal control group, an untreated HFSD group, and eight HFSD groups treated for 23 weeks with metformin, *C. citrinus* extract, the individual bioactive compounds, or a mixture of these compounds. We assessed body and organ weights, liver macroscopic features, serum markers of liver injury, oxidative stress biomarkers, histopathological changes, and hepatic fatty acid profiles. HFSD feeding induced significant body weight gain, hepatomegaly, severe tissue damage, elevated serum levels of aspartate aminotransferase (AST), alanine aminotransferase (ALT), gamma-glutamyl transferase (GGT), glucose and triglycerides, alongside heightened hepatic oxidative stress, and altered saturated fatty acid profiles. Conversely, treatment with metformin and the natural compounds attenuated HFSD-induced liver injury to varying degrees. Improvements in histopathological severity and serum liver enzymes correlated with decreased oxidative stress biomarkers. Notably, ellagic acid, gallic acid, d-limonene, and the bioactive mixture, significantly improved hepatic fatty acid profiles, whereas *p*-coumaric acid exhibited more modest effects. These results demonstrate that long-term administration of low-dose treatment can successfully improve all evaluated metabolic, biochemical, and histological parameter, suggesting that modulating oxidative stress and liver lipid composition is strongly linked to the mitigation of liver injury in this experimental MAFLD model.

## 1. Introduction

Global epidemiological data indicate that Metabolic dysfunction-associated fatty liver disease (MAFLD) affects about one-third of adults, and prevalence rates have increased in recent years alongside the global rise in obesity and metabolic disorders [1,2]. In Mexico, clinical and observational studies have reported a high prevalence of MAFLD, reaching nearly 50% among adults undergoing medical evaluation, often accompanied by obesity, dyslipidemia, and other metabolic comorbidities [3,4]. Recent evidence from Mexican populations confirms this high prevalence and its close association with metabolic risk factors [4].

Regional studies further underscore the magnitude of this condition. In central Mexico, including the municipality of Morelia, Michoacán, MAFLD prevalence has been reported to exceed 50% and is strongly associated with higher body mass index, highlighting obesity as a major determinant of hepatic steatosis in this population [5].

MAFLD involves excessive fat accumulation in the liver and encompasses a spectrum of liver changes, ranging from simple fatty liver to more severe conditions, including metabolic dysfunction–associated steatohepatitis, fibrosis, cirrhosis, and hepatocellular carcinoma [6]. Although early stages often show no symptoms, progressive MAFLD is linked to liver cell damage, metabolic disturbances, and an increased risk of liver and cardiovascular diseases [7,8].

Beyond lipid buildup, oxidative stress plays a crucial role in MAFLD progression and liver damage. When the liver accumulates lipids, it increases production of reactive oxygen species, which can damage cellular lipids and proteins. Both experimental and clinical research show that oxidative stress in MAFLD is often characterized by elevated levels of lipid peroxidation products, such as malondialdehyde (MDA) and 4-hydroxynonenal (4-HNE), as well as advanced oxidation protein products (AOPP) [9,10]. At the same time, alterations in paraoxonase-1 (PON1) activity, an enzyme critical for breaking down lipid peroxides and associated with high-density lipoproteins, are observed in diet-related metabolic issues and in changes in redox status, including total oxidant status (TOS), which are also present in MAFLD [11,12].

Metformin is widely used as a reference compound in experimental models of metabolic liver disease due to its reported hepatoprotective effects in both genetic and diet-induced settings. Experimental studies in rodents have shown that metformin reverses fatty liver changes and improves hepatic redox status, including reducing oxidative stress, under conditions of metabolic dysfunction [13,14].

Given the role of oxidative damage in MAFLD-related liver injury, there is growing interest in natural products with antioxidant and hepatoprotective properties as potential treatments. Most studies using plant extracts or compounds show protective effects against oxidative stress and liver damage in high-fat diet models, but use high doses for short periods; however, few studies use low doses over long periods [15,16]. *Callistemon citrinus* (Myrtaceae), a plant native to Australia and widely cultivated in Mexico, has attracted attention for its bioactive phytochemicals. In a previous study, we determined the terpenic composition of *C. citrinus* leaf extract using two-dimensional gas chromatography with time-of-flight mass spectrometry detection (GC × GC-ToFMS). The main terpenes were 1,8-cineole, d-limonene, and α-terpineol [17]. In another study, we reported that d-limonene reduced oxidative stress during hypercaloric diets [18].

Recently, we found ellagic acid, gallic acid, and *p*-coumaric acid in *C. citrinus* leaf extract using HPLC [19]. Finally, Garcia-Calderon reported that these phenolic acids could mitigate oxidative stress in the brains of Wistar rats fed a hypercaloric diet [20].

In other studies, ellagic acid has been shown to reduce hepatic steatosis and oxidative damage in experimental models [21,22], while gallic acid improves hepatic oxidative balance and regulates lipid levels in diet-induced steatosis [23,24,25]. Furthermore, *p*-coumaric acid exhibits antioxidant activity and protects against lipid oxidation in experimental contexts [26,27]. These compounds have been studied at doses of 50 to 200 mg/kg, but in the *C. citrinus* extract, they are present at lower concentrations than those previously reported. There are no existing studies on the use of *C. citrinus* to prevent liver damage from prolonged consumption of an HFSD. This research aimed to evaluate the effects of ethanolic leaf extract from *C. citrinus* and its major active compounds, d-limonene, gallic acid, ellagic acid, and *p*-coumaric acid, on liver injury, markers of oxidative stress, tissue pathology, and the fatty acid profile in a rat model of MAFLD induced by a high-fat–sucrose diet.

## 2. Results

### 2.1. Callistemon citrinus Extract, Its Major Compounds, and Metformin Reduce Weight Gain, Food Intake, and Organ Weights

**Figure 1:**
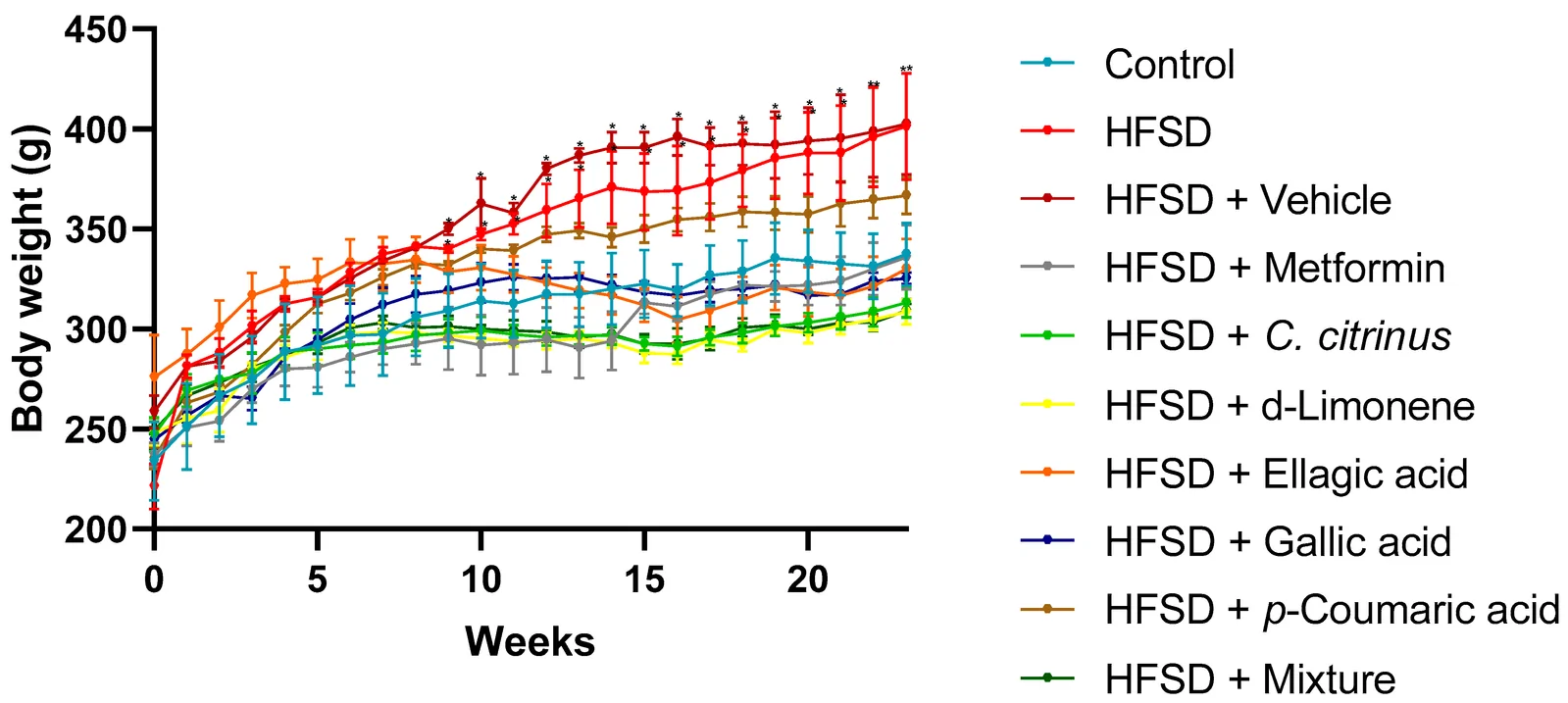
Illustrates weekly changes in body weight over the 23-week experiment. Wistar rats were weighed weekly after being fed either standard chow (control) or a high-fat, sucrose-rich diet (HFSD). The HFSD group received daily treatments of vehicle, metformin (20 mg/kg), *Callistemon citrinus* leaf extract (200 mg/kg), d-limonene (0.43 mg/kg), ellagic acid (0.0743 mg/kg), gallic acid (0.00694 mg/kg), *p*-coumaric acid (0.00047 mg/kg), or a combination of these bioactive compounds at the same doses. These treatments started at the beginning of the dietary intervention and continued throughout the 23-week period. Body weights are reported as mean ± SEM (*n* = 6 per group). Statistical analysis involved one-way ANOVA with Dunnett’s post-test, comparing each week’s results to the control; significant differences are marked with asterisks (*p* < 0.05).

Male Wistar rats on a high-fat–sucrose diet (HFSD) were treated with *Callistemon citrinus* extract (200 mg/kg), its main compounds, a combination of these compounds, or metformin (20 mg/kg). The weekly body weight progress over 23 weeks is shown in Figure 1. Rats fed HFSD showed a steady increase in weight compared with the control group, while vehicle-treated rats showed a similar pattern. However, treatment with metformin, *C. citrinus* ethanolic leaf extract, d-limonene, ellagic acid, gallic acid, and their combination reduced HFSD-induced weight gain, keeping body weights closer to the control. Among the phytochemicals tested, *p*-coumaric acid showed the least effect, with weight gain patterns similar to HFSD and HFSD + Vehicle groups. Significant differences from the control were observed in the later weeks, as shown in Figure 1.

As summarized in Table 1, HFSD-fed rats exhibited significantly greater body weight gain than the control group. HFSD feeding also significantly increased liver weight, epididymal fat weight, and liver index, whereas water intake did not differ significantly among the experimental groups. Vehicle administration did not modify body weight gain, food intake, organ weights, epididymal fat weight, or liver index compared with the HFSD group.

At the end of the study, metformin significantly reduced body weight gain and food intake compared with the HFSD group, and both liver weight and liver index were comparable to those of the control group. Likewise, rats treated with *C. citrinus* ethanolic leaf extract or *d*-limonene showed significantly lower body weight gain and food intake than HFSD-fed rats, accompanied by reduced liver weight, epididymal fat weight, and liver index. Ellagic acid, gallic acid, and the mixture also significantly reduced body weight gain relative to the HFSD group and were associated with lower liver weights. Ellagic acid and gallic acid produced the lowest liver index values among the treatment groups, whereas the mixture significantly reduced epididymal fat weight. In contrast, *p*-coumaric acid did not significantly reduce body weight gain or food intake compared with the HFSD group, although it significantly decreased liver weight and liver index. No significant differences were observed in heart or stomach weights among the experimental groups. Kidney weights were lower in several treatment groups than in the Control group but did not differ from those of HFSD-fed rats.

### 2.2. Effects of Callistemon citrinus, Its Major Compounds, and Metformin on the Macroscopic Liver Features

**Figure 2:**
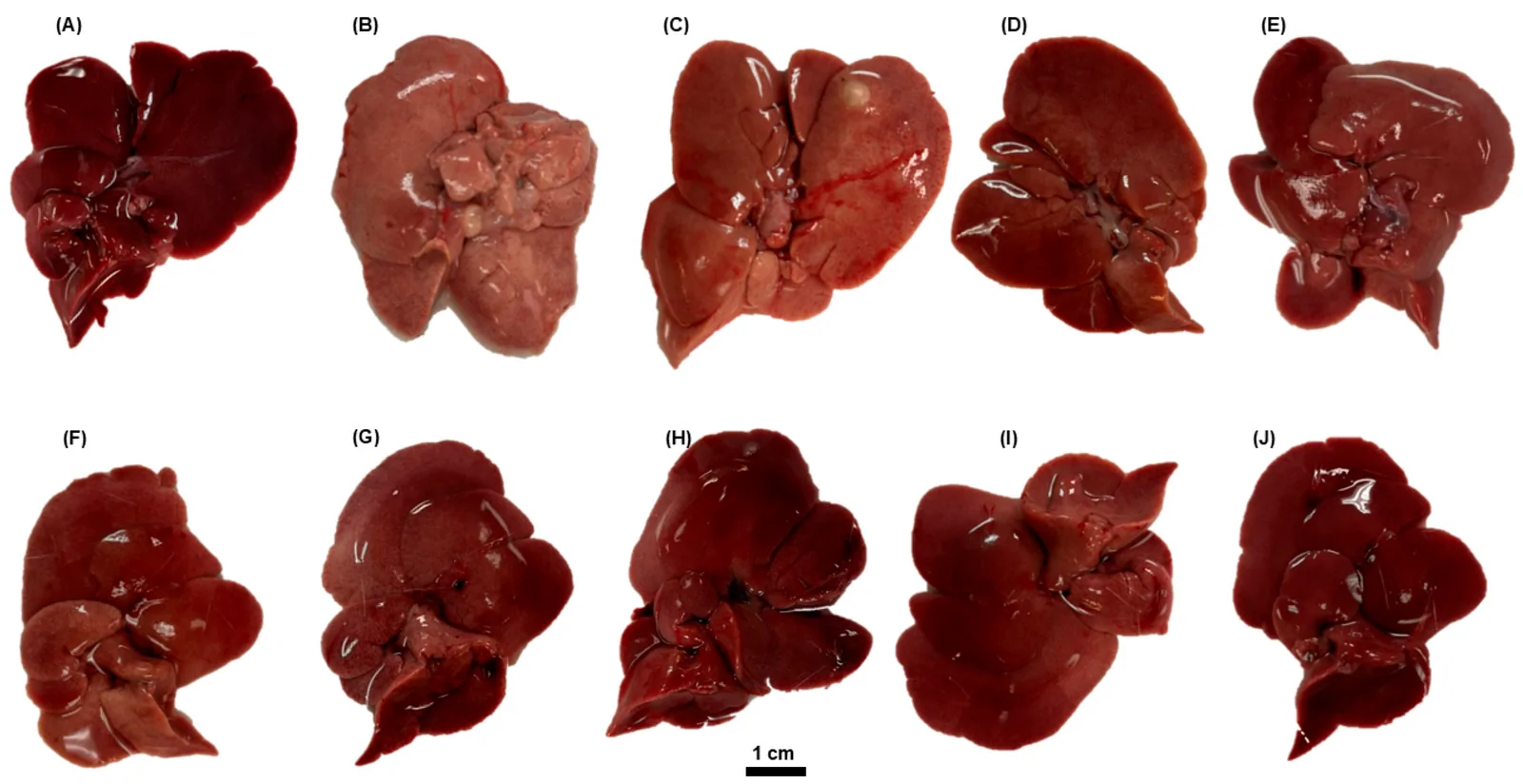
Representative macroscopic appearance of livers from rats with HFSD-induced metabolic dysfunction-associated steatotic liver disease (MASLD). (**A**) Control; (**B**) HFSD; (**C**) HFSD + vehicle; (**D**) HFSD + metformin (20 mg/kg); (**E**) HFSD + *Callistemon citrinus* ethanolic leaf extract (200 mg/kg); (**F**) HFSD + d-limonene (0.43 mg/kg); (**G**) HFSD + ellagic acid (0.0743 mg/kg); (**H**) HFSD + gallic acid (0.0069 mg/kg); (**I**) HFSD + *p*-coumaric acid (0.00047 mg/kg); and (**J**) HFSD + mixture (containing all compounds at the same doses used in the respective monotherapy groups). Representative livers were photographed at the end of the experimental period. Scale bar = 1 cm.

Macroscopic examination of the excised livers revealed clear differences among the experimental groups (Figure 2). Livers from control rats exhibited homogeneous dark-red coloration, a smooth capsular surface, distinct lobular anatomy, and sharply defined margins. Conversely, livers from HFSD-fed rats appeared pale and enlarged, with irregular surfaces and visible nodules. The HFSD + Vehicle group showed similar changes, indicating that vehicle administration did not alter the hepatic changes induced by HFSD. Rats treated with metformin had darker livers with fewer surface irregularities, consistent with the lower liver weight observed in this group (Table 1). Treatment with *Callistemon citrinus* ethanolic leaf extract produced a more uniform liver color and preserved lobular architecture compared with HFSD rats. D-limonene administration partially restored liver color and surface smoothness, although liver size remained larger than in controls, consistent with the liver weight data (Table 1). Ellagic acid and gallic acid treatments improved liver appearance, reducing pallor and smoothing the surface relative to HFSD rats. In contrast, livers from *p*-coumaric acid-treated rats were similar to those in the HFSD group, with increased size and uneven coloration. Notably, livers from rats given the bioactive compound mixture resembled the control group, showing a darker color and preserved surface structure.

### 2.3. Effects of Callistemon citrinus, Its Major Compounds, and Metformin on the Biochemical Parameters

**Figure 3:**
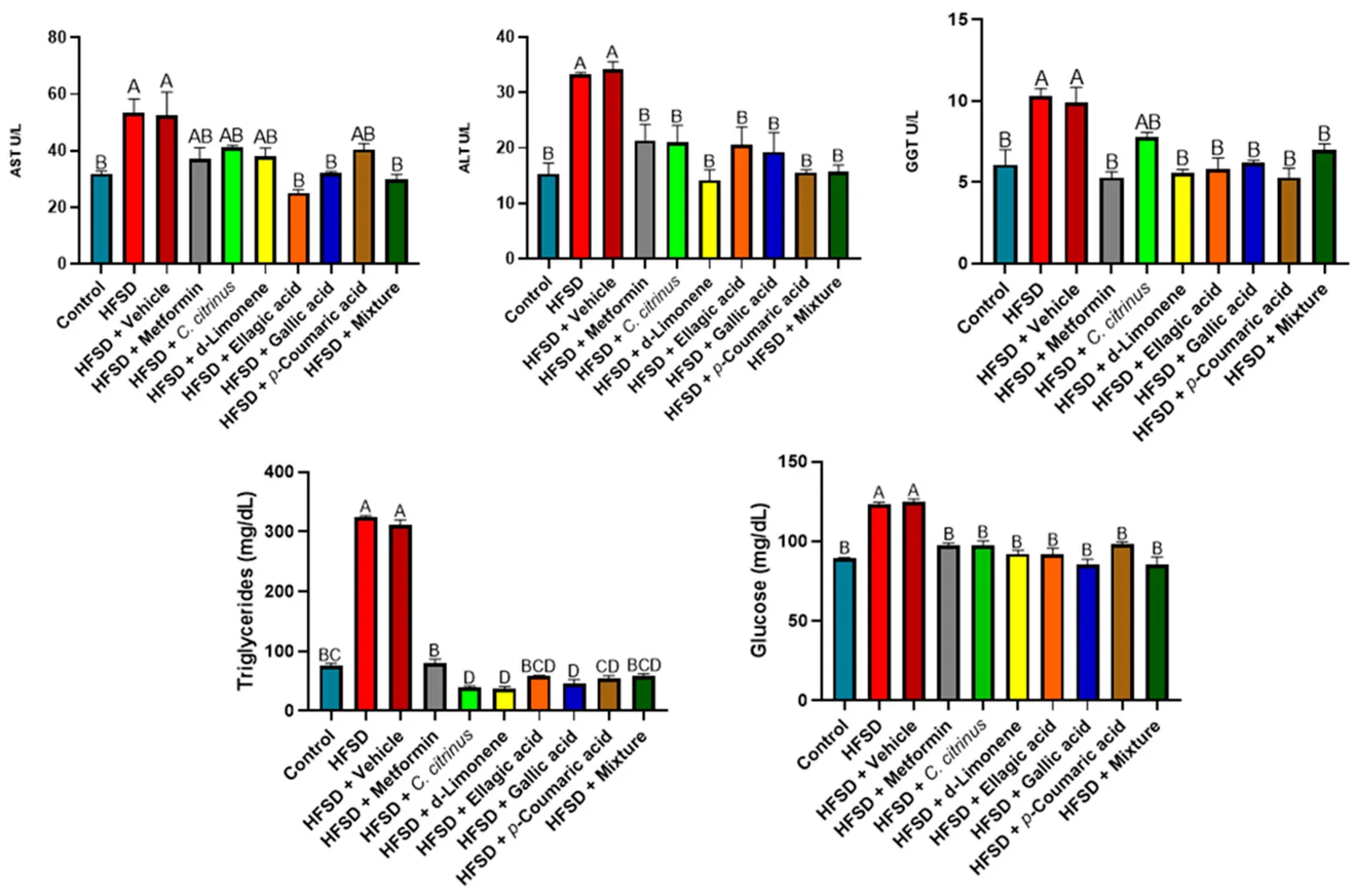
Biochemical markers of liver injury and metabolic alterations in rats with high-fat and sucrose diet (HFSD)-induced metabolic dysfunction-associated fatty liver disease. Serum activities of aspartate aminotransferase (AST), alanine aminotransferase (ALT), and gamma-glutamyl transferase (GGT), together with blood glucose and triglyceride concentrations, were determined in control rats and HFSD-fed rats receiving vehicle, metformin (20 mg/kg), *C. citrinus* leaf extract (200 mg/kg), d-limonene (0.43 mg/kg), ellagic acid (0.074 mg/kg), gallic acid (0.0069 mg/kg), *p*-coumaric acid (0.00047 mg/kg) or the bioactive compound mixture. Data are presented as mean ± SEM (*n* = 6). A, B, C and D indicate statistically significant differences among groups (one-way ANOVA followed by Tukey’s post hoc test, *p* < 0.05).

Biochemical parameters differed significantly among experimental groups (Figure 3). HFSD feeding increased AST, ALT, and GGT activities, as well as blood glucose and triglyceride concentrations, compared with the control group. Similar values were observed in the HFSD + Vehicle group. Metformin treatment significantly reduced ALT and GGT activities and lowered blood glucose and triglyceride concentrations compared with those of HFSD-fed rats. AST values showed an intermediate response and did not differ significantly from either the control or HFSD groups. Administration of *Callistemon citrinus* ethanolic leaf extract significantly reduced ALT activity and lowered blood glucose and triglyceride concentrations compared with the HFSD group, while AST and GGT values remained intermediate between those of control and HFSD rats. Treatment with d-limonene significantly lowered ALT activity and reduced blood glucose and triglyceride levels, while AST and GGT values showed no significant difference compared to either the control or HFSD groups. Ellagic acid, gallic acid, and the bioactive compound mixture significantly reduced AST, ALT, and GGT activities and decreased blood glucose concentrations compared with the HFSD group. Ellagic acid had a moderate effect on triglyceride levels, whereas gallic acid and the bioactive compound mixture lowered them compared with rats fed HFSD. *p*-Coumaric acid significantly decreased ALT activity and lowered blood glucose and triglyceride levels, but did not cause a notable change in AST compared to HFSD-fed rats. GGT levels in the *p*-coumaric acid group were similar to those in the control group.

### 2.4. Effects of Callistemon citrinus, Its Major Compounds, and Metformin on the Oxidative Stress Biomarkers

**Figure 4:**
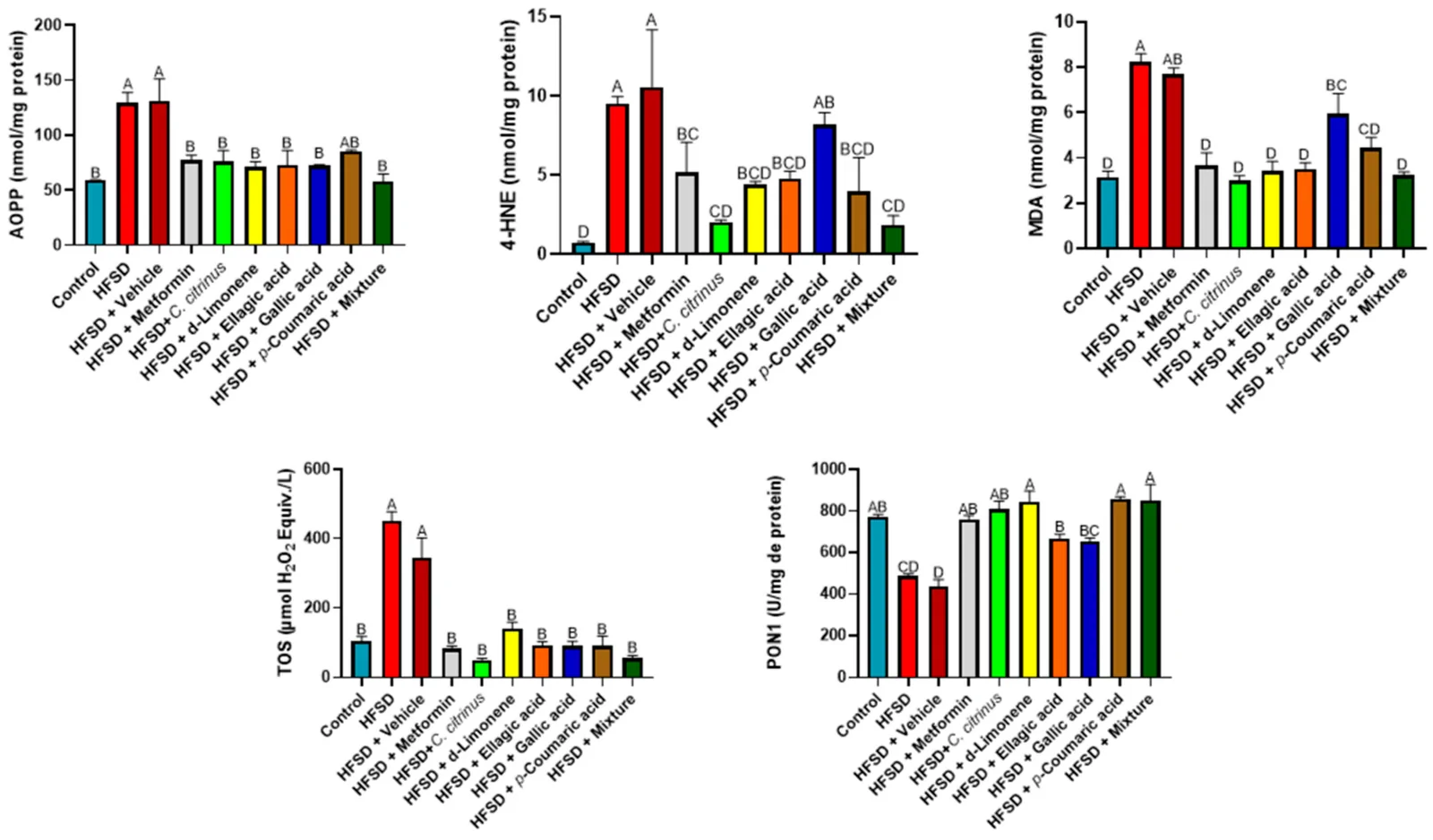
Biomarkers of oxidative stress, including AOPP, 4-HNE, MDA, TOS, and PON1 activity, were measured in liver homogenates from control and HFSD-fed rats receiving vehicle, metformin (20 mg/kg), *C. citrinus* leaf extract (200 mg/kg), d-limonene (0.43 mg/kg), ellagic acid (0.074 mg/kg), gallic acid (0.0069 mg/kg), *p*-coumaric acid (0.00047 mg/kg) or the bioactive compound mixture. Data are presented as mean ± SEM (*n* = 6). A, B, C and D indicate statistically significant differences among groups (one-way ANOVA followed by Tukey’s post hoc test, *p* < 0.05).

Oxidative stress biomarkers varied significantly among the experimental groups (Figure 4). HFSD feeding significantly increased hepatic AOPP, 4-HNE, MDA, and TOS levels compared with those of the control group. Meanwhile, PON1 activity was significantly lower in rats fed HFSD than in controls. Similar reductions were observed in the HFSD + Vehicle group, suggesting that vehicle administration did not alter the oxidative stress induced by HFSD. Metformin treatment significantly reduced hepatic AOPP, 4-HNE, MDA, and TOS levels compared with the HFSD group and restored PON1 activity to levels comparable to those of control rats. Administration of *C. citrinus* ethanolic leaf extract was also associated with significantly lower oxidative stress biomarkers and higher PON1 activity compared with HFSD-fed rats. Rats treated with d-limonene exhibited significant reductions in AOPP, 4-HNE, MDA, and TOS levels compared to the HFSD group and also showed increased PON1 activity. Similarly, treatments with ellagic and gallic acids significantly reduced markers of lipid and protein oxidation and partially restored PON1 activity compared with HFSD-fed rats. In contrast, treatment with *p*-coumaric acid only partially reduced oxidative stress biomarkers, as AOPP and MDA levels stayed higher than in control rats. PON1 activity in this group increased compared with HFSD-fed rats but did not reach the levels observed in controls. Notably, rats receiving the combination of bioactive compounds showed reductions in all oxidative stress biomarkers and a significant increase in PON1 activity, with values closely resembling those of the control group.

### 2.5. Effects of Callistemon citrinus, Its Major Compounds, and Metformin on the Histopathological Evaluation of Liver Tissue

**Figure 5:**
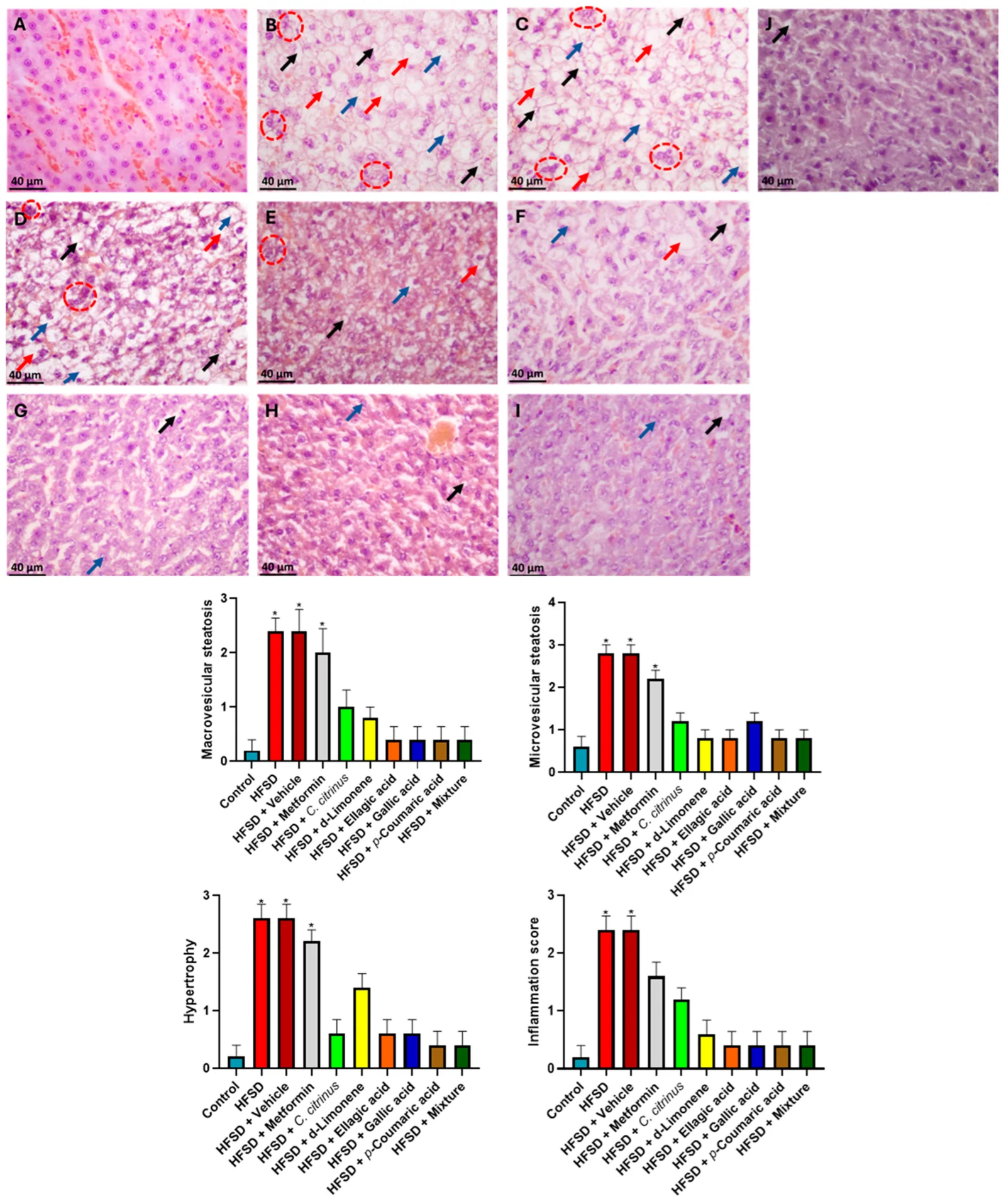
Representative histological micrographs of hematoxylin and eosin-stained liver sections from the experimental groups. (**A**) control; (**B**) high-fat and sucrose diet (HFSD); (**C**) HFSD + vehicle; (**D**) HFSD + metformin (20 mg/kg); (**E**) HFSD + *C. citrinus* ethanolic leaf extract (200 mg/kg); (**F**) HFSD + d-limonene (0.43 mg/kg); (**G**) HFSD + ellagic acid (0.074 mg/kg); (**H**) HFSD + gallic acid (0.0069 mg/kg); (**I**) HFSD + *p*-coumaric acid (0.00047 mg/kg); and (**J**) HFSD + mixture. Macrovesicular steatosis (black arrows): large lipid droplets within hepatocytes. Microvesicular steatosis (blue arrows): small lipid droplets within hepatocytes. Hepatocellular hypertrophy (red arrows): enlarged hepatocytes relative to surrounding cells. Inflammatory cell aggregates (red dashed circles): clusters of inflammatory cells within the hepatic parenchyma. Scale bar = 40 µm, * *p* < 0.05.

Histological analysis of hematoxylin and eosin-stained liver sections revealed normal liver architecture in the control group, with no evidence of steatosis, hepatocellular hypertrophy, or inflammatory infiltrates (Figure 5). Conversely, livers from rats fed HFSD and HFSD + vehicle displayed widespread macrovesicular steatosis (characterized by large lipid droplets displacing the hepatocytes’ nuclei) and microvesicular steatosis (shown by multiple small lipid droplets in the cytoplasm). This lipid accumulation was accompanied by hepatocellular hypertrophy (enlarged hepatocytes) and clusters of inflammatory cell aggregates within the hepatic parenchyma.

Semi-quantitative scoring confirms that the HFSD and HFSD + Vehicle groups had significantly higher pathological scores across all four parameters, macrovesicular steatosis, microvesicular steatosis, hypertrophy, and inflammation, compared to controls (*p* < 0.05; Figure 5). Metformin treatment failed to rescue the liver from HFSD-induced architectural damage. The histopathological parameters were similar to those of the untreated HFSD group. The metformin treatment group exhibited scores that significantly exceeded those of the control group, demonstrating weak efficacy in reversing direct hepatic steatosis and parenchymal inflammation.

Treatment with *C. citrinus* leaf extract and its component d-limonene resulted in a moderate reduction in liver injury. Both treatments reduced macrovesicular and microvesicular steatosis scores compared with the HFSD group. However, d-limonene exhibited a slightly higher hypertrophy score than the *C. citrinus* extract, indicating that although lipid clearance was effective, some cellular swelling remained under d-limonene treatment.

Ellagic acid, gallic acid, and *p*-coumaric acid showed the strongest therapeutic response, with the lowest histopathological scores among the intervention groups. In these animals, liver tissue displayed minimal steatosis, fewer inflammatory spots, and reduced hepatocellular hypertrophy. These histological characteristics, including macrovesicular and microvesicular steatosis, hypertrophy, and inflammation, were similar to those observed in the control group (see Figure 5). Finally, the mixture group retained baseline scores for steatosis, hypertrophy, and inflammation, confirming that the combination effectively prevents MAFLD progression caused by dietary fat and sucrose.

### 2.6. Effects of Callistemon citrinus, Its Major Compounds, and Metformin on the Hepatic Fatty Acid Profile

The hepatic fatty acid profiles showed significant variation across the experimental groups (Table 2). The HFSD group and the HFSD + vehicle group exhibited high levels of saturated fatty acids; however, the level of palmitic acid (C16:0) was lower than that of stearic acid (C18:0). In contrast, treatment with *C. citrinus*, d-limonene, gallic acid, *p*-coumaric acid, ellagic acid, the mixture, and metformin resulted in higher levels of C16:0 compared to C18:0. All treatment groups showed the presence of monounsaturated fatty acids (MUFA) and polyunsaturated fatty acids (PUFAs), with the exception of C22:4, C22:5, and C22:6, which were not detected in the metformin group. The D-limonene and metformin groups shared similarities, as they had a higher level of palmitoleic acid (C16:1) than the other treatment groups; however, D-limonene showed an increase in levels of γ-linolenic acid (C18: 3), eicosatrienoic acid (C20:3), and arachidonic acid (C20:4) compared with the metformin group. Conversely, in the control group, the fatty acid levels were lower than in the treatment groups.

### 2.7. Analysis of the Effects of Callistemon citrinus, Its Major Compounds, and Metformin on Lipid Metabolism, Membrane Fluidity and Inflammation

To evaluate the metabolic impact of a high-fat, high-sucrose diet (HFSD) and subsequent treatments on lipid synthesis, stearoyl-CoA desaturase-1 (SCD-1) indices for C18 and C16 lipogenesis were measured. The untreated HFSD and vehicle (HFSD + V) groups exhibited severe metabolic disruption, resulting in upstream or downstream polyunsaturated fatty acid (PUFA) levels that fell below the analytical detection limit (0.00*; Table 3). In contrast, pharmacological and experimental interventions effectively rescued these lipogenic pathways. The control group maintained a baseline C18 lipogenesis index of 0.41. Introduction of the positive control, metformin (HFSD + MET), led to a marked increase in the C18 index to 3.13. Similar trends of upregulated C18 lipogenesis were observed across the experimental treatment groups, with HFSD + G (2.76), HFSD + E (2.71), and HFSD + LIM (2.62) showing the highest values among the single compounds, while HFSD + *C.c* demonstrated a more moderate recovery (1.16). Conversely, the C16 lipogenesis index remained tightly regulated and relatively uniform across all active treatment cohorts, ranging from 0.02 (HFSD + E) to 0.05 (HFSD + MET).

The activity of Δ-5-desaturase (D5D), a crucial enzyme in PUFA biosynthesis, was completely suppressed or undetectable in the HFSD and HFSD + V groups (0.00*). All the treatments successfully reactivated this pathway, surpassing the control baseline, which remained non-detectable in this dataset. Metformin treatment increased D5D activity to 15.50. Remarkably, the HFSD + G (29.94) and HFSD + E (22.43) groups showed the strongest increases in D5D activity, far exceeding the levels seen with metformin. The other treatment groups (MIX, *C.c*, LIM, and p-CA) displayed similar D5D activity levels, ranging from 17.06 to 18.50. The ratio of saturated fatty acids to monounsaturated fatty acids (SFA/MUFA) served as an indicator of cellular membrane fluidity, with a lower ratio suggesting greater fluidity. Because raw PUFA data were missing, this ratio could not be calculated for the untreated HFSD and HFSD + V groups. The healthy control group showed an SFA/MUFA ratio of 5.11. All therapeutic treatments significantly reduced this ratio compared to the control. The lowest ratio—indicating the highest membrane fluidity—was observed with metformin (0.98), followed closely by HFSD + MIX (1.13), HFSD + G (1.12), and HFSD + E (1.14). The HFSD + *C.c* group had the highest ratio among the treatments at 1.94, though it still represented a substantial decrease from the control baseline.

Finally, the ARA/DHA ratio was measured to assess systemic inflammation, as shown in Table 3. The control group had an inflammatory index of 6.82. This index could not be calculated for the HFSD, HFSD + V, and HFSD + MET groups due to undetectable levels of key PUFA components (0.00*). Among the treatment groups, HFSD + E showed a notable anti-inflammatory effect, lowering the ARA/DHA ratio to 5.92, even below the control level. Groups treated with MIX (6.76), *C.c* (6.87), G (6.53), and p-CA (6.87) maintained an inflammatory index close to the healthy control. In contrast, LIM treatment worsened inflammation, resulting in a significantly higher index of 10.07.

## 3. Discussion

In this study, long-term HFSD feeding led to significant metabolic and hepatic dysfunction, as evidenced by increased body weight, elevated blood triglyceride levels as serum markers of liver damage and oxidative stress, notable histopathological changes, and altered hepatic fatty acid profiles. This confirms the successful creation of a diet-induced model of metabolic dysfunction–related fatty liver disease. Generally, high-fat, high-sucrose diets (HFSD) trigger hyperphagic cycles of overeating by desensitizing central satiety pathways, leading to a loss of metabolic self-regulation in animals. However, specific bioactive compounds can disrupt this pathology via distinct gut-to-brain signaling or peripheral metabolic mechanisms. For instance, gallic acid crosses the blood–brain barrier, exerting robust neuroprotective and antioxidant effects; it mitigates hypothalamic oxidative stress and neuroinflammation, thereby clearing chemical blockades that obstruct leptin receptors (Ob-Rb) in the arcuate nucleus [28]. Similarly, ellagic acid limits weight gain and lowers overall caloric intake by acting as a localized pancreatic lipase inhibitor in the gut lumen [29], while d-limonene improves central leptin sensitivity [17]. *p*-Coumaric acid does not alter cumulative food intake under high-energy dietary conditions; rather, it mitigates adiposity and hepatic steatosis through peripheral pathways, namely by suppressing lipogenic enzyme expression and increasing fecal lipid excretion [30]. In contrast to these phytochemical mechanisms, metformin regulates energy balance through a distinct neuroendocrine axis, upregulating circulating Growth Differentiation Factor 15 (GDF15) to suppress appetite via hindbrain receptors [31]. When evaluated in vivo, oral monotherapies with metformin, *Callistemon citrinus* extract, and their isolated bioactive components differentially mitigated HFSD-induced pathology across multiple physiological levels. These interventions systematically limited pathological weight gain and normalized serum liver enzyme levels. At the hepatic level, therapeutic benefits included a marked reduction in oxidative stress markers, partial recovery of paraoxonase-1 (PON1) activity, preservation of normal liver histology, and favorable restructuring of lipid profiles.

High-fat and high-sucrose diets are commonly used in experimental research to induce obesity-related metabolic changes and liver injury because they mimic key features of metabolic dysfunction–related fatty liver disease in rodents, including excessive weight gain, hepatic steatosis, and biochemical signs of liver damage [32]. Consistent with this approach, long-term HFSD feeding in this study resulted in systemic and hepatic phenotypes that align with diet-induced metabolic liver dysfunction, as evidenced by metabolic, biochemical, histopathological, and lipidomic changes.

Diet-induced hepatic injury in experimental models is closely linked to lipid oversupply and lipotoxicity, which promote hepatocellular stress and impair liver function independently of neutral triglyceride accumulation. When male Wistar rats consume a high-fat–sucrose diet (HFSD), their livers become overwhelmed by an excess of fatty acids and carbohydrates, specifically glucose and fructose. This influx accelerates triglyceride synthesis via de novo lipogenesis, ultimately leading to dyslipidemia, visceral fat accumulation, and hepatic steatosis (fatty liver disease). However, treatments involving metformin or the primary bioactive compounds found in *Callistemon citrinus* extract, d-limonene, gallic acid, ellagic acid, and *p*-coumaric acid can mitigate these adverse metabolic effects through distinct, complementary mechanisms. For instance, d-limonene effectively modulates lipid accumulation in both hepatic and adipose tissues by upregulating peroxisome proliferator-activated receptor alpha (PPAR-α), thereby enhancing the transcription of genes responsible for clearing circulating triglycerides, and by suppressing adipogenesis [33]. Gallic and ellagic acids significantly reduce the expression of FAS and SREBP-1c while restoring the secretion of adiponectin, an adipose-derived hormone that improves insulin sensitivity and stimulates tissue-level triglyceride clearance. Additionally, these two acids reduce malondialdehyde (MDA) levels, restore superoxide dismutase (SOD) activity, and protect mitochondrial integrity, thereby promoting efficient fat oxidation [34]. Meanwhile, *p*-coumaric acid enhances glucose and lipid uptake in skeletal muscle and the liver by improving insulin signaling (e.g., IRS-1) and activating AMPK [35]. Because *Callistemon citrinus* extract naturally combines all of these compounds, it exerts a powerful pleiotropic effect on lipid metabolism. Finally, metformin serves as a robust control by activating AMPK and suppressing SREBP-1c, thereby preventing the conversion of carbohydrates into triglycerides and upregulating β-oxidation [36].

The central role of non-esterified saturated fatty acids and other lipotoxic lipid species in driving hepatocellular injury and disease progression has been established in translational studies of fatty liver disease [37]. In this context, the elevation of serum transaminases and GGT observed under HFSD conditions in the present study is consistent with hepatocellular injury patterns commonly reported in diet-induced models of metabolic liver disease.

Histopathological analysis showed that HFSD feeding resulted in steatosis and hepatocellular ballooning, lesions widely recognized as defining features of fatty liver-associated liver injury in both experimental models and human disease [38]. Detailed histopathological analyses indicate that these alterations reflect structural correlates of metabolic stress and hepatocellular vulnerability rather than isolated morphological changes [39]. Accordingly, the histological phenotype observed in the HFSD group supports the validity of the present model for studying diet-induced liver injury.

Oxidative stress plays a key role in the development of fatty liver disease in both experimental and clinical settings. Mitochondrial dysfunction is linked to increased oxidative stress and greater vulnerability of liver cells as the disease progresses [40]. At the same time, antioxidant systems such as paraoxonase-1 (PON1) are considered important markers of oxidative imbalance in metabolic liver conditions [41]. Consistent with these findings, the rise in liver oxidative stress markers and the decrease in PON1 activity in rats fed a high-fat, high-sugar diet (HFSD) support the characteristic features of metabolic liver impairment.

In addition to biochemical and histological damage, feeding with HFSD was linked to changes in liver fatty acid composition. Lipidomic studies of liver tissue have shown that fatty liver disease involves increased levels of saturated fatty acids and shifts in polyunsaturated and long-chain fatty acid species, which are associated with cellular stress and disease severity [42]. Notably, liver biopsies from patients with steatohepatitis reveal the accumulation of saturated fatty acids and a redistribution of unsaturated fatty acids [43]. Moreover, research indicates that the types of dietary fatty acids and metabolic overload influence hepatic fatty acid profiles during metabolic stress [44]. The fatty acid profile observed in the HFSD group in this study aligns with lipidomic changes reported in the liver during metabolic liver disease.

Overall, these findings show that prolonged HFSD feeding causes a complex form of liver injury, involving metabolic disturbances, hepatocellular damage, oxidative stress, alterations in tissue structure, and hepatic lipid remodeling. This combined phenotype provides a useful experimental model for testing drugs and plant-based compounds to reduce oxidative stress and liver metabolic damage.

Pharmacological and phytochemical interventions targeting oxidative stress and hepatocellular injury are central strategies for attenuating diet-induced liver damage. In the present study, oral administration of metformin, *Callistemon citrinus* ethanolic leaf extract, and selected bioactive compounds differentially mitigated HFSD-induced liver injury, as evidenced by reductions in serum transaminase and GGT levels and increases in glucose and triglycerides, which serve as oxidative stress biomarkers.

Metformin has demonstrated benefits in managing type 2 diabetes and could also be effective against MASLD. This effectiveness stems from its capacity to decrease hepatic gluconeogenesis and inhibit enzymes involved in fatty acid synthesis. Moreover, it offers antioxidant benefits by lowering lipid peroxidation [13,14]. Clinical evidence further supports beneficial associations between ongoing metformin use and liver-related outcomes in patients with metabolic disease [45]. In our study, the metformin dose was lower, and the duration of administration was longer than in other studies, owing to gastrointestinal side effects [46]. Our findings indicated that prolonged low-dose metformin therapy was associated with decreased serum markers of liver damage and diminished hepatic oxidative stress compared with rats fed a high-fat, high-sugar diet (HFSD).

In the metformin treatment group, the liver’s fatty acid profile showed higher palmitic acid levels than stearic acid. Additionally, palmitoleic and oleic acid levels increased, while linoleic and gamma-linolenic acid levels decreased. This may be because metformin activates the AMPK pathway, which inhibits the SREBP-1c transcription factor, the main driver of desaturase activity [47]. Such inhibition significantly reduces GLA levels, potentially affecting membrane fluidity. Histologically, this is evident as macro- and microvesicular steatosis and hypertrophy, in contrast to the outcomes observed in treatments involving *C. citrinus* and its major compounds.

Among the bioactive compounds studied, gallic acid has been shown to reduce steatotic liver disease and oxidative stress in the livers of rodents fed a high-fat diet, underscoring its role as a phenolic antioxidant in diet-induced liver damage [18,48]. Likewise, ellagic acid has been shown to reduce oxidative stress and improve liver biochemical markers in experimental models of hepatic injury, supporting its role as a hepatoprotective polyphenol under metabolic stress [49].

For d-limonene, direct experimental evidence from a rat model fed a hypercaloric (high-fat–sucrose) diet shows that this terpene reduces hepatic oxidative stress and mitigates diet-induced liver injury associated with metabolic dysfunction [18]. The current findings support these observations and suggest that oral administration of gallic acid, ellagic acid, and d-limonene can partially alleviate HFSD-induced oxidative imbalance and hepatocellular injury.

Paraoxonase-1 (PON1) activity provides an additional link between oxidative stress and liver injury, as reduced PON1 activity has been associated with increased oxidative burden and impaired liver function in metabolic liver disease [41]. The partial recovery of hepatic PON1 activity observed in treated groups suggests that reducing oxidative stress may help enhance hepatic antioxidant capacity.

Notably, the mixture of bioactive compounds showed the most consistent pattern of improvement across serum liver enzymes and hepatic oxidative stress biomarkers. Combined phytochemical formulations are proposed to increase antioxidant coverage through complementary actions among individual components, potentially enhancing overall protection against oxidative damage without implying the pharmacological additive effects experimentally demonstrated [50].

Overall, these results show that reducing oxidative stress is strongly associated with improvements in biochemical indicators of liver damage, whether through drug- or plant-based treatments. The varying responses across compounds highlight the importance of both individual and combined effects in influencing HFSD-induced liver problems.

Histopathological changes observed in HFSD-fed rats matched the metabolic and biochemical problems caused by long-term exposure to a high-fat–sucrose diet. HFSD intake led to increased body weight, higher liver and fat tissue weights (Figure 1, Table 1), and notable liver abnormalities, including hepatomegaly, pale color, and surface irregularities (Figure 2). These visible changes were accompanied by severe microscopic damage, including widespread steatosis, hepatocellular ballooning, and disruption of liver structure, as evidenced by significantly higher histopathological scores than in the control group (Figure 5). These structural features are common signs of diet-induced metabolic dysfunction-related fatty liver disease in both experimental and clinical settings [39,51,52].

The observed structural liver damage in HFSD-fed rats was strongly associated with biochemical markers of liver injury and oxidative stress. HFSD feeding significantly increased serum AST, ALT, GGT, glucose, and triglyceride levels (Figure 3), indicating liver cell damage and impaired liver function. At the same time, levels of oxidative stress markers, including AOPP, 4-HNE, MDA, and TOS, increased markedly, whereas PON1 activity decreased (Figure 4). Oxidative stress is widely acknowledged as a central factor in the progression of fatty liver disease, linking metabolic overload, injury to liver cells, and changes at the tissue level [53,54,55].

Treatment with metformin and most evaluated bioactive compounds clearly reduced HFSD-induced liver injury, both macroscopically and microscopically. These groups showed decreased liver weight (Table 1), better gross liver appearance (Figure 2), and a significant reduction in histopathological damage, including less steatosis and hepatocellular ballooning (Figure 4). Similar findings of histological recovery, aligned with biochemical improvements, have been reported in experimental models of fatty liver disease following reductions in oxidative stress and metabolic injury [56,57,58].

The histological improvements observed in the treated groups were also associated with normalization of serum liver enzymes and reductions in oxidative stress biomarkers. Also, showed the greatest decreases in lipid peroxidation and protein oxidation markers, along with partial or full recovery of PON1 activity (Figure 4). This strong correlation suggests a link between oxidative stress levels and the extent of liver damage, consistent with existing models of MAFLD progression [53,54,57].

In contrast, *p*-coumaric acid treatment showed a different response pattern. Although it significantly reduced liver weight compared with HFSD-fed rats (Table 1), it did not significantly affect body weight gain or food intake and was still associated with ongoing macroscopic and microscopic liver changes. Consequently, rats treated with *p*-coumaric acid still exhibited residual steatosis and structural disruption (Figure 5), along with incomplete normalization of oxidative stress markers and serum transaminases (Figure 3 and Figure 4). These results suggest that its protective effect at the tissue level is more limited than that of other treatments.

The strong correlation among liver weight, macroscopic appearance, histopathological severity, serum markers of liver injury, and hepatic oxidative stress highlights the link between biochemical dysfunction and structural damage in this HFSD-induced MAFLD model. Combining histological evaluation with biochemical profiling provides a comprehensive approach to assess therapeutic effectiveness in experimental MAFLD models and aligns with current guidelines for thorough preclinical evaluation [39,56].

HFSD feeding caused significant changes in hepatic fatty acid composition compared with the control group, marked by a notable increase in saturated fatty acids, especially palmitic acid (C16:0) and stearic acid (C18:0), and the disappearance of detectable polyunsaturated and very-long-chain fatty acids (Table 2). Similar changes in liver lipid composition have been consistently observed in experimental models of diet-induced fatty liver disease and in human NAFLD/MASLD, where excess dietary lipids and carbohydrates increase saturated fatty acids and decrease long-chain polyunsaturated fatty acids in the liver [42,57,59].

In this study, unexpected results were obtained, as it is commonly reported that palmitic acid (C16:0) increases during lipogenesis on a high-fat, high-sugar diet [60]. However, the increase occurred in stearic acid (C18:0) in the group fed the high-fat–sucrose diet solely. The observed increase in stearic acid may be attributed to increased elongase 6 activity, which converts C16:0 (palmitic acid) to C18:0 (stearic acid) to mitigate palmitic acid-induced lipotoxicity [61]. Additionally, the rise in stearic acid in the absence of oleic acid suggests that desaturase 1 (SCD1), which introduces double bonds into fatty acids, is either inhibited or that the diet is high in saturated fatty acids [62]. The diet’s fatty acid profile, as documented by Magaña-Rodríguez et al. [63], showed high levels of saturated fatty acids and trans C18:1. Consequently, both the HFSD and HFSD + vehicle groups contained only saturated fatty acids. However, groups fed an HFSD and treated with different compounds exhibited varying concentrations of monounsaturated fatty acids (MUFAs) and polyunsaturated fatty acids (PUFAs), indicating the activity of these compounds.

D-limonene and metformin treatments displayed similarities, with high levels of C16:1 and C18:1 fatty acids. Other treatments also contained these acids but had lower levels of C16:1 relative to C18:1. Notably, only the d-limonene group exhibited increased levels of C18:2, gamma-C18:3, and C20:4 fatty acids and showed the highest fatty acid diversity. These findings show that d-limonene not only reduces fat buildup and TGA levels, as previously documented, but also modulates fatty acids in the liver, inducing a metabolic response because not only does oleic acid neutralize the toxicity of palmitic acid, but its high concentrations of gamma-linolenic acid protect cell membranes, maintaining their fluidity, unlike metformin.

In this study, enrichment of C16:0 and C18:0 in HFSD-fed rats was associated with severe histopathological damage and elevated oxidative stress markers in liver tissue. The accumulation of saturated fatty acids in steatotic livers is closely linked to hepatocellular stress and lipotoxic traits in fatty liver disease, providing an important pathological context for understanding the liver injury observed under prolonged metabolic stress [57,64].

Additionally, the absence of detectable arachidonic acid (C20:4) and other long-chain fatty acids in HFSD-fed rats suggests a significant disruption of hepatic fatty acid composition, consistent with lipidomic studies of human liver tissue that show a progressive loss of PUFA pools as MAFLD severity increases [42]. Vehicle administration did not alter the hepatic fatty acid pattern induced by HFSD, indicating that the observed changes were diet-driven. Metformin partially modified this profile, as evidenced by reduced C18:0 levels compared with those in HFSD-fed rats and the reappearance of γ-linolenic acid and arachidonic acid (Table 2). Reports indicate partial recovery of unsaturated fatty acids in experimental models of fatty liver disease following interventions that enhance metabolic control and reduce liver injury. This observation supports the idea that changes across different endpoints are interconnected. However, it does not necessarily mean that the same lipidomic mechanisms are involved [56,59].

Among the tested phytochemical treatments, *Callistemon citrinus* ethanolic leaf extract was more similar to the control group than the other treatments. However, it contained a higher amount of PUFA than the control group (Table 2). The fatty acid profile shows a pattern similar to that of the phenolic acid treatment. These compositional changes were accompanied by significant improvements in histopathological features and oxidative stress markers, highlighting a link between hepatic lipid composition and liver structural integrity in experimental fatty liver disease [65].

Notably, *p*-coumaric acid treatment, which has the lowest phenolic concentration used during long-term treatment, showed limited recovery of unsaturated or very-long-chain species (Table 2). This pattern aligns with the modest improvements observed in histopathological and biochemical endpoints in this group and underscores the variability among interventions in hepatic lipid remodeling.

The mixture treatment exhibits a fatty acid profile similar to that of all the treatment groups. This hepatic fatty acid pattern corresponded to the most significant overall improvement across liver outcome areas observed in this study, supporting a coordinated link between hepatic lipid composition and liver health.

Our analysis of SCD-1 index values across different groups shows a clear pattern: lipid toxicity leading to collapse, followed by therapeutic rescue. The control group maintained an average index of 0.41, indicating normal lipid desaturation balance. In contrast, the 23-week HFSD group showed an index near zero, signaling a total blockade of SCD-1 activity. This significant decline indicates a severe lipotoxic state, in which the tissue’s ability to convert saturated fatty acids (C18:0) into healthier monounsaturated fatty acids (C18:1*n*-9) was completely suppressed by excessive dietary lipids. Our treatments effectively reversed the metabolic blockage, raising the SCD-1 index to between 1.0 and 3.0. This increase does not signify harmful de novo lipogenesis but rather a protective, compensatory response. By significantly increasing SCD-1 activity, the treatments helped tissues quickly clear accumulated lipotoxic stearic acid (C18:0) and convert it into oleic acid (C18:1*n*-9). This process actively restores membrane fluidity and shields cells from ongoing diet-related lipotoxic stress [66].

The two Δ-9-desaturase indices showed distinct patterns across the experimental groups. The C18:1*n*-9/C18:0 index showed significant therapeutic improvement, whereas the C16:1*n*-7/C16:0 index remained near zero in the untreated HFSD group and stayed low (~0.03) in both the control and treatment groups. This difference aligns with the known enzyme kinetics of SCD-1, which has a much higher affinity for 18-carbon saturated fatty acids than for 16-carbon substrates. The complete suppression of the C16 index to zero in the HFSD group underscores severe substrate competition and feedback inhibition under chronic lipid overload. The treatments restored the C16 index to the healthy control group’s exact physiological baseline (~0.03) and selectively upregulated the C18 index to clear lipotoxic stearic acid, thereby demonstrating a targeted, homeostatic therapeutic mechanism [60]. This pattern confirms that the treatments successfully alleviated lipotoxic membrane rigidity without triggering aberrant, pathological de novo lipogenesis pathways.

Overall, these findings indicate that a low, prolonged dose of these compounds activates multiple mechanisms that enhance PUFA synthesis and reduce lipotoxicity caused by saturated fatty acids. The correlation among hepatic fatty acid profiles, histopathological severity, and oxidative stress markers underscores the importance of liver lipid profiling as a comprehensive measure for evaluating liver injury and recovery in this HFSD-induced MAFLD model.

Beyond the observed changes in hepatic fatty acid composition, the protective effects of the bioactive compounds under study likely involve broader metabolic, antioxidant, and immunomodulatory pathways. Recent experimental findings show that D-limonene alleviates diet-induced metabolic dysfunction and fatty liver disease by inhibiting the PPARγ/SCD-1 pathway, thereby reducing hepatic lipid accumulation and promoting lipid metabolic balance. Specifically, D-limonene was found to lower PPARγ and SCD-1 expression, and supporting cellular and pharmacological studies suggest that this signaling pathway is key to its lipid-lowering effects [67]. This understanding provides valuable context for interpreting changes in fatty acid composition and SCD-1-related desaturation indices observed in animals treated with D-limonene in this study. However, because SCD-1 levels or activity were not directly assessed, these indices should be viewed as proxy indicators of hepatic fatty acid desaturation, not direct measures of SCD-1 activity.

Oxidative and inflammatory signaling may represent additional mechanisms that contribute to the hepatoprotective response. Lemon-derived nanovesicles have been shown to activate the AhR/Nrf2 pathway, promote Nrf2 nuclear localization, reduce reactive oxygen species production, and reduce inflammatory cell migration in experimental models [68]. More recently, studies in human hepatocytes and in rats fed a high-fat diet demonstrated that these nanovesicles reduced oxidative stress and increased hepatic Nrf2 and HO-1 expression, supporting the role of Nrf2-dependent antioxidant defenses in metabolically induced liver injury [69]. Although lemon-derived nanovesicles differ biologically from the isolated compounds examined here, these findings suggest that citrus-associated bioactive molecules can influence redox-sensitive signaling pathways. This aligns with the significant reductions in AOPP, 4-HNE, MDA, and TOS, as well as the recovery of PON1 activity observed after several treatments.

These metabolic and redox effects can be understood within the emerging framework of the spleen–liver axis. The spleen serves as a key reservoir of immune cells that can influence hepatic inflammation and fibrosis. Studies have shown that splenic CD11b + CD43Hi Ly6C monocytes are more likely to migrate to injured liver tissue, differentiate into macrophages, and help activate hepatic stellate cells, thereby promoting fibrosis [70]. This immune cell population has also been found to expand in both the spleen and the liver under metabolically stressful dietary conditions, suggesting that spleen-derived immune cells may contribute to chronic, diet-induced liver damage. Pro-inflammatory mediators such as TNF-α and IL-6, along with redox-related signaling pathways, likely facilitate communication between the spleen and the liver. Therefore, reducing oxidative stress and inflammation with bioactive compounds might help modulate this interorgan immune-metabolic interaction.

Recently, Lan et al. [71] demonstrated that limonin reduces inflammation and fibrogenesis by inhibiting the STAT3 pathway, which affects galectin-3/mTORC1 signaling. However, it remains unclear whether these pathways are directly influenced by *C. citrinus* itself or by the specific compounds examined in this study. Additionally, our experimental design did not include direct assessments of splenic immune cell populations, cytokine trafficking, Nrf2 signaling, PPARγ/SCD-1 expression, or the STAT3/galectin-3/mTORC1 pathway. Future research that combines molecular pathway analyses with studies of immune cell movement in the spleen will be essential to determine whether modulating the spleen–liver axis plays a role in the sustained liver protection observed in this study.

The reported results in this study are limited to young Wistar rats. Due to the Bioethics Committee’s recommendation to use fewer animals, we were unable to conduct a study with mature animals to assess the effects of the *C. citrinus* extract and its main compound on MALFD. It would be interesting to conduct studies with female rats to determine whether this diet, as used in this study, causes similar liver damage. Another limitation is the inability to perform pharmacokinetic studies to determine whether the hepatoprotective activity arises from the original compounds or from biotransformed products. Future research on the effects of compounds from *C. citrinus* on signaling pathways involved in MAFLD would help clarify their mechanisms of action. In addition, isobologram analysis or fixed-ratio combinations with multiple dose gradients would be desirable to assess synergistic effects.

## 4. Materials and Methods

### 4.1. Chemicals and Reagents

All reagents used in this study were analytical grade. The bioactive compounds evaluated as treatments included R-(+)-limonene (97% purity; Cat. No. 183164), gallic acid (Cat. No. G7384), ellagic acid hydrate (Cat. No. 372749), and *p*-coumaric acid (98% purity, HPLC grade; Cat. No. C9008), all obtained from Sigma-Aldrich (St. Louis, MO, USA). The ethanolic leaf extract of *C. citrinus* was prepared and concentrated using a rotary evaporator (Büchi, Rotavapor^®^ R-210; Büchi Labortechnik AG, Flawil, Switzerland).

Reagents used in lipid peroxidation assays included 1-methyl-2-phenylindole, butylated hydroxytoluene (BHT), methanesulfonic acid, and hydrochloric acid. Paraoxonase-1 (PON1) activity was determined using 4-nitrophenyl acetate, Tris–HCl buffer, and calcium chloride. Advanced oxidation protein products (AOPPs) were quantified using potassium iodide, glacial acetic acid, chloramine-T, and phosphate buffer (20 mM, pH 7.4). Total oxidative status (TOS) was assessed using xylenol orange, ferrous ammonium sulfate, 3,3′,5,5′-tetramethylbenzidine (TMB), sodium chloride, and glycerol, with hydrogen peroxide as a calibration standard.

Histological tissue processing was performed with an automated tissue processor (Microm, model STP 120, Walldorf, Germany), and paraffin-embedded tissue blocks were sectioned with a rotary microtome (Microm, model HM 325, Walldorf, Germany). Liver tissue homogenization was performed in phosphate buffer.

Spectrophotometric measurements were performed using a UV–Vis spectrophotometer (Thermo Spectronic, Genesys™ 10, Rochester, NY, USA). Serum biochemical parameters, including aspartate aminotransferase (AST), alanine aminotransferase (ALT), and gamma-glutamyl transferase (GGT), were determined spectrophotometrically using commercial enzymatic colorimetric kits (Spinreact, Naucalpan de Juárez, Mexico; a subsidiary of Toyobo, Girona, Spain).

The hepatic fatty acid profile was determined by gas chromatography–mass spectrometry (GC–MS) using an HP-5MS capillary column (5% phenyl methylsiloxane, 60 m × 0.25 mm × 0.25 μm; Agilent Technologies, Santa Clara, CA, USA) coupled to a quadrupole mass spectrometer (Hewlett-Packard 5975C, Santa Clara, CA, USA) operating under electron impact ionization.

### 4.2. Plant Material and Preparation of the Ethanolic Extract

Leaves from four-year-old *C. citrinus* (Curtis) Skeels (Myrtaceae) plants were collected in Morelia, Michoacán, Mexico. Plant material was taxonomically identified, and a voucher specimen (EBUM23538) was deposited at the Biology School of Universidad Michoacana de San Nicolás de Hidalgo. The leaves were air-dried at room temperature and macerated in 96% ethanol at a 1:10 (*w*/*v*) ratio for five days with periodic agitation. The extract was filtered through Whatman No. 1 filter paper, and the solvent was removed under reduced pressure on a rotary evaporator at 45 °C. The extraction yield was approximately 20% (*w*/*w*). The resulting crude extract was stored at −20 °C until use. Extract preparation and standardization followed the previously reported methodology [17].

### 4.3. Animals

Male Wistar rats (two months old; 180–200 g) were obtained from the animal facility at the Instituto de Investigaciones Químico Biológicas, Universidad Michoacana de San Nicolás de Hidalgo (UMSNH). Animals were housed in plastic cages under controlled conditions (12 h light/dark cycle, 23–24 °C, 60–70% relative humidity) with free access to standard chow and water.

All experimental procedures were approved by the Animal Bioethics Committee of UMSNH (protocol ID: IIQB-CIBE-06-2024; approval date: 12 January 2024) and conducted in accordance with the Mexican Official Standard for the care and use of laboratory animals (NOM-062-ZOO-1999).

### 4.4. Experimental Design and HFSD-Induced MAFLD Model

Animals were randomly assigned to ten experimental groups (*n* = 6 per group) using a simple randomization procedure. The study lasted 23 weeks. MAFLD was induced by chronic exposure to an HFSD prepared by supplementing standard rodent chow with vegetable fat (20.83%), pork lard (20.83%), and sucrose (16.68%).

**Figure 6:**
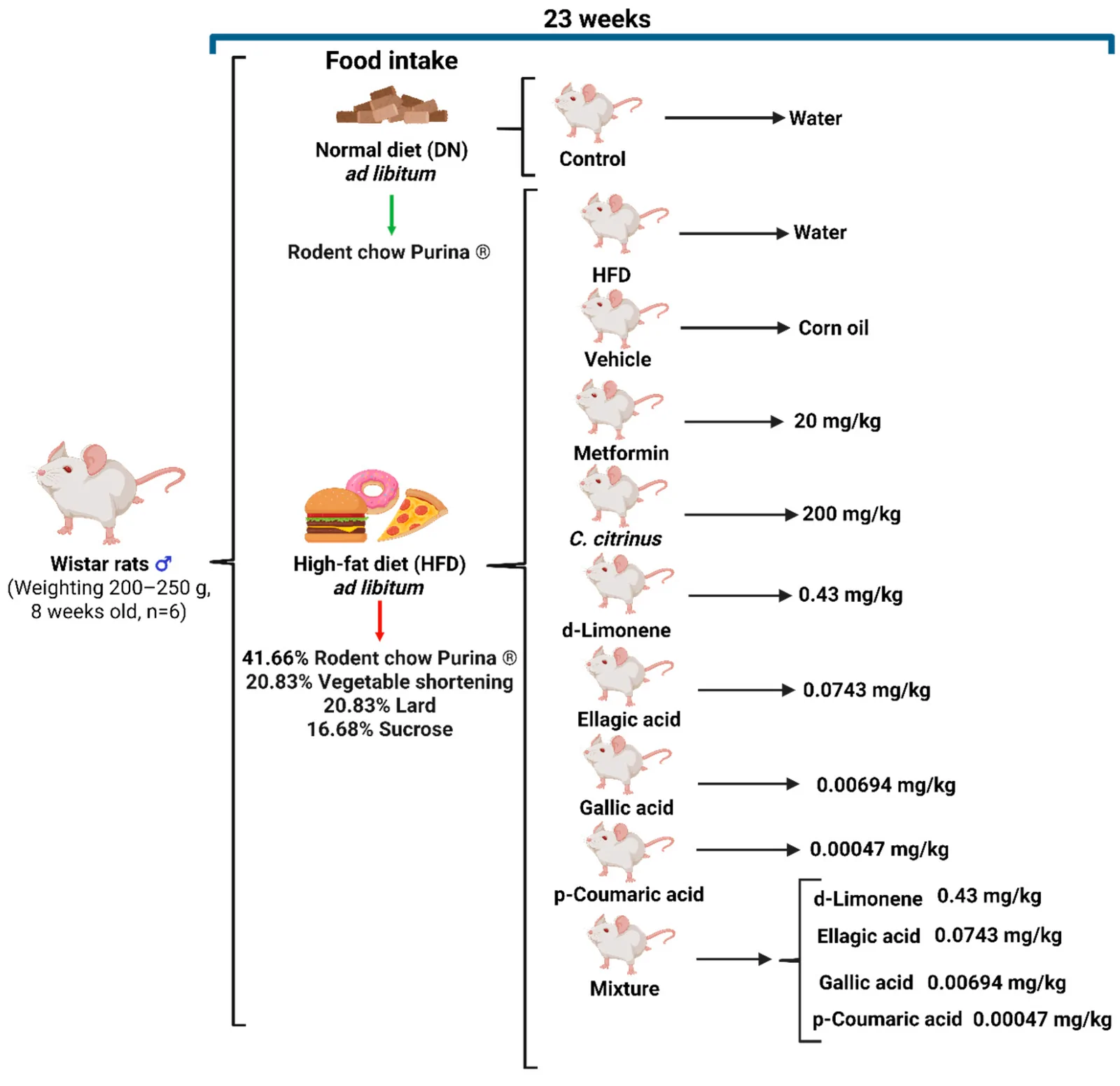
Experimental design. Male Wistar rats were maintained for 23 weeks on either standard rodent chow or a high-fat and sucrose diet (HFSD) and allocated to ten experimental groups (*n* = 6 per group). Water, vehicle, metformin, *C. citrinus* ethanolic leaf extract, individual phytochemicals, or their mixture were administered daily by oral gavage.

The experimental groups were defined as follows: Group 1, control, was fed with a standard rodent chow (Purina^®^ Rodent Chow) and administered water once daily by oral gavage (1 mL/kg body weight); Group 2, HFSD, fed with a HFSD plus water by oral gavage at the same volume; Group 3, HFSD + Vehicle, administered corn oil once daily by oral gavage (1 mL/kg body weight); Group 4, HFSD + Metformin (20 mg/kg); Group 5, HFSD + *C. citrinus* ethanolic leaf extract (200 mg/kg); Group 6, HFSD + d-limonene (0.43 mg/kg); Group 7, HFSD + ellagic acid (0.0743 mg/kg); Group 8, HFSD + gallic acid (0.00694 mg/kg); Group 9, HFSD + *p*-coumaric acid (0.00047 mg/kg); and Group 10, HFSD + Mixture, containing all compounds at their respective individual doses. The doses used in this study are based on those reported in a previous study [20]. We chose this experimental design over a traditional synergistic matrix because diet-induced obesity and liver damage are complex, multifactorial conditions involving lipid peroxidation, protein oxidation, and insulin resistance. Each compound targets distinct pathways; combining them enables comprehensive, multi-target coverage. All treatments began alongside dietary intervention and were administered once daily via oral gavage at a fixed volume of 1 mL/kg body weight, with weekly adjustments. Dosing occurred at 9:00 a.m. in the home cage throughout the entire 23-week experiment. Water served as the vehicle for both the control and HFSD groups, whereas corn oil was used for all lipophilic treatments. No rats died during the experiments (Figure 6).

At the end of 23 weeks, the animals underwent a 12–13 h fast before being deeply anesthetized with sodium pentobarbital (150 mg/kg, intraperitoneally). Whole blood was collected via cardiac puncture under deep anesthesia. After confirming death, liver tissue and white adipose tissue depots, including the retroperitoneal, epididymal, mesenteric, and inguinal fat pads, were quickly removed, rinsed in cold saline, snap-frozen, and stored at −80 °C for subsequent analysis.

### 4.5. Biochemical Parameters

Serum activities of aspartate aminotransferase (AST), alanine aminotransferase (ALT), and gamma-glutamyl transferase (GGT) were measured in serum samples using commercial enzymatic colorimetric kits (Spinreact, Mexico; subsidiary of Toyobo, Spain), according to the manufacturer’s instructions. Absorbance was recorded using a spectrophotometer, and enzyme activities were expressed as U/L. Blood glucose and triglyceride concentrations were determined immediately after blood collection using an Accutrend Plus^®^ analyzer (Roche Diagnostics, Mannheim, Germany) and specific reagent strips for each analyte. Measurements were performed in fresh whole blood according to the manufacturer’s instructions, and results were expressed as mg/dL.

### 4.6. Histopathological Evaluation of Liver Tissue

Liver samples were rinsed in cold saline solution (0.9%), fixed in 10% neutral-buffered formalin for at least 24 h, and processed for paraffin embedding. Paraffin blocks were sectioned at 4 µm thickness and stained with hematoxylin and eosin (H&E) according to standard histological procedures. Histopathological evaluation was performed in a blinded manner using the rodent NAFLD scoring system described by Liang et al. [72]. Macrovesicular steatosis, microvesicular steatosis, hepatocellular hypertrophy, and inflammatory cell aggregates were assessed by light microscopy. Steatosis and hypertrophy were graded according to the percentage of hepatic parenchyma affected as follows: 0 (<5%), 1 (5–33%), 2 (34–66%), and 3 (>66%). These parameters were evaluated at 40–200× magnification, considering only hepatocyte sheets and excluding portal tracts and terminal hepatic venules. Inflammation was assessed at 100× magnification by determining the average number of inflammatory foci in five microscopic fields. A focus was defined as a cluster of at least five inflammatory cells. All histological analyses were performed by a trained observer unaware of experimental group allocation.

### 4.7. Liver Tissue Homogenization

Liver tissue samples (0.5 g) were homogenized in 1 mL of ice-cold 50 mM phosphate buffer (pH 7.4). Homogenates were centrifuged at 12,000× *g* for 20 min at 4 °C. Supernatants were collected and stored at −80 °C until further biochemical analyses. Protein concentration was determined by the Bradford method [73].

### 4.8. Oxidative Stress Biomarkers

#### 4.8.1. Lipid Peroxidation: Malondialdehyde (MDA) and 4-Hydroxynonenal (4-HNE)

Levels of malondialdehyde (MDA) and 4-hydroxynonenal (4-HNE) were measured using a colorimetric method based on their condensation with 1-methyl-2-phenylindole [74]. For this purpose, 200 µL of liver homogenate was combined with 650 µL of 10 mM 1-methyl-2-phenylindole and 5 µL of 5 mM butylated hydroxytoluene (BHT). The reaction was initiated by adding 150 µL of concentrated hydrochloric acid or methanesulfonic acid, then allowed to proceed at 45 °C for 60 min. After incubation, samples were cooled, and absorbance was measured at 586 nm. Data were normalized to tissue weight and expressed as nmol/g tissue.

#### 4.8.2. Paraoxonase-1 (PON1) Activity

Paraoxonase-1 (PON1) activity was evaluated by measuring the hydrolysis rate of 4-nitrophenyl acetate, as previously described [75]. Liver homogenate (5 µL) was added to 1 mL of a reaction mixture containing 25 mM Tris–HCl buffer (pH 8.0), 10 mM CaCl_2_, and 1 mM 4-nitrophenyl acetate. The increase in absorbance was monitored at 402 nm at 30 s intervals for 3 min to determine enzymatic activity. A blank without homogenate was used to correct for non-enzymatic substrate hydrolysis. PON1 activity was normalized to protein content and expressed as µmol/min/mg protein.

#### 4.8.3. Advanced Oxidation Protein Products (AOPP)

Advanced oxidation protein products (AOPP) were determined using a spectrophotometric assay based on iodide-mediated oxidation, following a previously established method [76]. Liver homogenate (50 µL) was mixed with 1 mL of 20 mM phosphate buffer (pH 7.4), 50 µL of 1.16 M potassium iodide, and 100 µL of acetic acid. After a 2 min reaction at room temperature, absorbance was measured at 340 nm. AOPP concentrations were calculated from a chloramine-T standard curve and expressed as chloramine-T equivalents.

#### 4.8.4. Total Oxidative Status (TOS)

Total oxidative status (TOS) was determined using a modified ferrous ion–xylenol orange (FOX) assay, which is based on the oxidation of ferrous ions to ferric ions by oxidant species in the sample, followed by formation of a colored complex with xylenol orange under acidic conditions [77]. A fixed volume of liver homogenate 140 µL was mixed with an acidic reagent containing 150 µM xylenol orange, 140 mM NaCl, 1.35 M glycerol, and sulfuric acid. The initial absorbance was recorded as the sample blank. The reaction was initiated by adding a ferrous ammonium sulfate–based reagent supplemented with 3,3′,5,5′-tetramethylbenzidine (TMB) as a chromogenic enhancer. The mixture was incubated at room temperature until a stable endpoint was reached (approximately 4 min), and absorbance was measured at 560 nm. TOS values were calculated using a hydrogen peroxide standard curve and expressed as µmol H_2_O_2_ equivalents per liter.

### 4.9. Total Lipid Extraction, Fatty Acid Methyl Ester (FAME) Preparation, and GC–MS Analysis in Rat Liver Tissue

Hepatic tissue was handled under refrigerated conditions to preserve lipid integrity. Briefly, 0.1 g of liver tissue was mechanically disrupted in a chilled mortar with 2 mL of a chloroform–methanol mixture (2:1, *v*/*v*). The resulting suspension was transferred to centrifuge tubes and clarified by centrifugation at 13,000 rpm for 10 min at 4 °C. The recovered extract was subjected to liquid–liquid partitioning by adding 100 µL of distilled water, then vigorously mixing and centrifuging at 3000 rpm for 20 min at 4 °C. After separation, the lower organic layer was collected and stored at −20 °C until derivatization.

Fatty acid methyl esters were produced from 100 µL of the organic extract via alkaline transesterification. Samples were combined with 1 mL of 0.5 N methanolic sodium hydroxide and heated at 100 °C for 60 min. After cooling, methylation was completed by adding 1 mL of boron trifluoride in methanol, followed by incubation at 100 °C for an additional 20 min. Once the reaction mixture reached room temperature, phase extraction was performed by adding 2 mL of distilled water and 3 mL of hexane. Samples were vortexed for 20 s and allowed to separate. The hexane layer was collected, and the extraction was repeated with an additional 3 mL of hexane. The combined organic fractions were evaporated to dryness under a nitrogen stream and then reconstituted in isooctane prior to instrumental analysis [17].

Chromatographic separation of FAMEs was carried out using an Agilent 7890A gas chromatograph (Agilent Technologies, Santa Clara, CA, USA) equipped with an HP-5MS capillary column (5% phenyl methylsiloxane; 60 m × 0.25 mm internal diameter, 0.25 µm film thickness) and interfaced with a quadrupole mass spectrometer operating under electron impact ionization (Hewlett-Packard 5975C, Santa Clara, CA, USA). The oven temperature was initially held at 60 °C for 1 min, then increased to 280 °C at 8 °C/min. The injector, ion source, and quadrupole temperatures were set to 230 °C, 230 °C, and 150 °C, respectively. Helium was used as the carrier gas at a constant flow rate of 1 mL/min. Samples were injected (1 µL) in split mode (10:1). Mass spectra were recorded in EI mode at 70 eV over a mass-to-charge ratio (*m*/*z*) range of 50–500, with a detector voltage of −1737 V.

Chromatographic data were processed using MSChem software (Version E.02.01.1177, Agilent Technologies). Fatty acid identification was achieved by spectral matching against entries from the Wiley 275 and the National Institute of Standards and Technology (NIST, version 2.0) libraries. Fatty acid levels were expressed as relative peak area percentages. Quantitative analysis was performed using methyl decanoate (C10:0 FAME) as an external calibration standard.

Because measuring direct mRNA expression or protein levels of these enzymes under multiple dynamic experimental treatments is often highly restrictive, product-to-precursor fatty acid ratios derived from quantitative lipid profiling are widely accepted as functional, empirical proxies of actual in vivo desaturase and elongase kinetics [78,79]. To evaluate changes in lipid metabolism across the 10 experimental groups, lipid enzyme activity proxies were manually calculated as product-to-precursor fatty acid ratios derived from the GC/MS quantitative profile. Specifically, Δ-9-desaturase activity was estimated using the ratio of C18:1*n*-9/C18:0, while elongase activity was calculated using the ratio (C18:0 + C18: 1*n*-9)/(C16:0 + C16: 1*n*-7), following the established literature protocols [73]. These ratios serve as a robust empirical proxy for in vivo enzyme kinetics, providing a clear, downstream readout of functional physiological adaptation across our distinct experimental conditions.

### 4.10. Statistical Analysis

Data are presented as mean ± standard error of the mean (SEM). Statistical analyses were performed using one-way analysis of variance (ANOVA). When a significant overall effect was detected, Tukey’s honestly significant difference (HSD) test was used for multiple comparisons among groups. Statistical analyses were conducted using JMP Pro 13, and graphical representations were generated with GraphPad Prism V8.0. Differences were considered statistically significant at *p* < 0.05.

## 5. Conclusions

This study demonstrates that a long-term, low-dose therapeutic approach using *Callistemon citrinus* leaf extract and its key phytochemicals effectively counteracts the multifaceted pathology of HFSD-induced MAFLD in rats. Beyond mitigating classic markers of systemic metabolic stress, hepatic lipid accumulation, and parenchymal inflammation, interventions with d-limonene, gallic acid, ellagic acid, and the bioactive mixture successfully reversed hepatic lipotoxicity. This protective effect was driven by a distinct structural remodeling of the liver’s fatty acid composition, characterized by a significant reduction in saturated fatty acid pools (palmitic and stearic acids) and the preservation of crucial polyunsaturated fatty acid (PUFA) networks that are required for membrane fluidity. The varying degrees of efficacy—contrasting the profound protection of the bioactive mixture with the modest effects of *p*-coumaric acid—underscore the importance of individual compound dynamics in lipid homeostatic pathways. Ultimately, these results highlight that sustained, low-dose antioxidant and lipidomic modulation is a viable paradigm for halting the progression of metabolic liver injury.

## Figures and Tables

**Table 1:** Body weight gain, food intake, and organ weights in HFSD-fed rats receiving the indicated treatments.

Group	Weight Gain (g)	Food Intake (g/rat/day)	Liver (g)	Heart (g)	Kidney (g)	Epididymal Fat (g)	Stomach (g)	Liver Index (%)
Control	97.33 ± 4.67 ^b^	21.02 ± 0.22 ^a^	10.63 ± 0.83 ^b^	1.16 ± 0.13 ^a^	1.98 ± 0.18 ^a^	8.95 ± 2.09 ^c^	1.70 ± 0.19 ^a^	3.09 ± 0.08 ^cd^
HFSD	154.67 ± 16.51 ^a^	22.33 ± 0.61 ^a^	13.76 ± 0.44 ^a^	1.34 ± 0.08 ^a^	2.06 ± 0.13 ^a^	30.98 ± 3.93 ^a^	1.58 ± 0.08 ^a^	4.03 ± 0.20 ^a^
HFSD + Vehicle	153.33 ± 26.64 ^a^	22.47 ± 0.64 ^a^	14.04 ± 1.16 ^a^	1.24 ± 0.09 ^a^	2.10 ± 0.12 ^a^	25.37 ± 0.64 ^ab^	1.45 ± 0.06 ^a^	3.90 ± 0.09 ^ab^
HFSD + Metformin (20 mg/kg)	52.67 ± 3.33 ^c^	13.64 ± 0.34 ^b^	10.46 ± 0.12 ^b^	1.06 ± 0.04 ^a^	1.95 ± 0.15 ^a^	18.69 ± 3.07 ^bc^	1.49 ± 0.16 ^a^	3.35 ± 0.08 ^bc^
HFSD + *C. citrinus* (200 mg/kg)	80.00 ± 2.00 ^b^	11.35 ± 0.37 ^c^	9.07 ± 0.46 ^b^	1.04 ± 0.09 ^a^	1.70 ± 0.12 ^a^	19.00 ± 2.72 ^bc^	1.26 ± 0.06 ^a^	3.34 ± 0.09 ^bc^
HFSD + d-Limonene (0.43 mg/kg)	53.33 ± 2.91 ^c^	10.84 ± 0.37 ^c^	9.17 ± 0.57 ^b^	1.20 ± 0.12 ^a^	1.87 ± 0.08 ^a^	15.04 ± 1.28 ^bc^	1.27 ± 0.06 ^a^	3.27 ± 0.09 ^cd^
HFSD + Ellagic acid (0.074 mg/kg)	54.67 ± 3.33 ^c^	12.84 ± 0.44 ^c^	8.90 ± 0.55 ^b^	1.13 ± 0.03 ^a^	1.88 ± 0.12 ^a^	22.38 ± 3.46 ^ab^	1.27 ± 0.04 ^a^	2.81 ± 0.08 ^cd^
HFSD + Gallic acid (0.0069 mg/kg)	63.33 ± 4.37 ^c^	11.72 ± 0.36 ^c^	9.05 ± 0.40 ^b^	1.11 ± 0.02 ^a^	2.00 ± 0.12 ^a^	23.13 ± 2.11 ^ab^	1.35 ± 0.10 ^a^	2.74 ± 0.13 ^d^
HFSD + *p*-Coumaric acid (0.00047 mg/kg)	131.33 ± 4.06 ^ab^	21.35 ± 0.41 ^a^	8.85 ± 0.23 ^b^	1.24 ± 0.03 ^a^	2.17 ± 0.10 ^a^	15.39 ± 3.95 ^bc^	1.41 ± 0.07 ^a^	2.98 ± 0.05 ^cd^
HFSD + Mixture	56.00 ± 1.16 ^c^	12.23 ± 0.38 ^c^	9.49 ± 0.25 ^b^	1.20 ± 0.08 ^a^	2.04 ± 0.13 ^a^	18.70 ± 0.88 ^bc^	1.39 ± 0.11 ^a^	2.88 ± 0.17 ^cd^

Data are shown as mean ± SEM (*n* = 6). Statistical comparisons were made using one-way ANOVA with Tukey’s HSD post hoc test (*p* < 0.05). Letters ^a^, ^b^, ^c^ and ^d^ denote statistically significant differences between the control, HFSD, and treatment groups.

**Table 2:** Percentage of major fatty acids in the livers of control rats and the different treatments.

Fatty Acid	C	HFSD	HFSD + V	HFSD + MET	HFSD + MIX	HFSD + *C.c*	HFSD + LIM	HFSD + G	HFSD + E	HFSD + p-CA
C14:0, Myristic	0.59 ± 0.02 abc	0.34 ± 0.01 d	0.58 ± 0.04 abc	0.66 ± 0.08 abc	0.50 ± 0.02 cd	0.70 ± 0.05 abc	0.36 ± 0.05 d	0.75 ± 0.15 a	0.71 ± 0.00 ab	0.52 ± 0.22 bcd
C15:0, Pentadecylic	0.20 ± 0.09	0.14 ± 0.00	0.13 ± 0.02	n.d	n.d	0.13 ± 0.14	0.23 ± 0.00	0.19 ± 0.02	0.25 ± 0.01	n.d
C16:0, Palmitic	8.92 ± 3.33 d	11.90 ± 1.81 cd	12.87 ± 2.38 cd	16.76 ± 3.04 ab	19.01 ± 1.72 ab	8.75 ± 0.02 d	19.89 ± 0.55 a	16.71 ± 3.23 ab	19.80 ± 0.57 a	15.34 ± 0.97 bc
C17:0, Margaric	0.51 ± 0.20 ab	0.36 ± 0.02 b	0.59 ± 0.17 a	0.55 ± 0.00 ab	0.58 ± 0.02 ab	0.44 ± 0.07 ab	0.64 ± 0.07 a	0.54 ± 0.03 ab	0.57 ± 0.02 ab	0.53 ± 0.17 ab
C18:0, Stearic	9.37 ± 0.07 bcd	15.11 ± 0.46 a	17.30 ± 0.95 a	7.94 ± 0.66 e	10.41 ± 0.67 b	7.54 ± 0.36 e	10.00 ± 0.28 b	8.59 ± 0.52 bc	9.73 ± 1.46 bc	10.57 ± 0.42 b
C20:0, Arachidic	n.d	n.d	n.d	0.21 ± 0.18	n.d	n.d	0.14 ± 0.03	0.06 ± 0.01	0.07 ± 0.01	0.04 ± 0.01
C16:1*n*-9, Palmitoleic	0.27 ± 0.12	n.d	n.d	0.82 ± 0.07	0.56 ± 0.09	0.37 ± 0.00	0.72 ± 0.24	0.44 ± 0.00	0.48 ± 0.36	0.41 ± 0.05
C18:1*n*-9, Oleic	3.82 ± 1.29	n.d	n.d	24.88 ± 3.42	25.90 ± 2.68	8.74 ± 0.48	26.16 ± 2.35	23.73 ± 6.22	26.36 ± 0.50	18.76 ± 3.63
C20:1*n*-9, Eicosenoic	n.d	n.d	n.d	0.32 ± 0.02	0.37 ± 0.09	n.d	0.39 ± 0.03	n.d	n.d	n.d
C18:2*n*-6, Linoleic	10.42 ± 0.96	n.d	n.d	6.75 ± 0.07	10.87 ± 0.60	5.98 ± 0.04	9.16 ± 1.03	7.86 ± 1.37	9.44 ± 0.26	8.50 ± 1.42
C20:2*n*-6, Eicosadienoic	n.d	n.d	n.d	0.11 ± 0.00	0.10 ± 0.00	n.d	0.12 ± 0.00	n.d	n.d	n.d
C18:3*n*-3, Linolenic	n.d	n.d	n.d	0.35 ± 0.08	0.55 ± 0.17	0.11 ± 0.00	1.02 ± 0.04	0.63 ± 0.25	0.54 ± 0.02	0.28 ± 0.10
C20:3*n*-6, DGLA	n.d	n.d	n.d	0.16 ± 0.06	0.42 ± 0.06	0.33 ± 0.07	0.33 ± 0.00	0.17 ± 0.04	0.28 ± 0.06	0.45 ± 0.01
C20:4*n*-6. Arachidonic	8.12 ± 1.23	n.d	n.d	2.48 ± 0.70	7.77 ± 1.49	5.63 ± 0.49	5.74 ± 2.25	5.09 ± 0.97	6.28 ± 1.46	8.11 ± 1.00
C22:5*n*-3, DPA	n.d	n.d	n.d	n.d	0.93 ± 0.16	0.29 ± 0.02	0.59 ± 0.14	0.50 ± 0.19	0.54 ± 0.24	0.42 ± 0.16
C22:6*n*-3, DHA	1.19 ± 0.52	n.d	n.d	n.d	1.15 ± 0.16	0.82 ± 0.09	0.57 ± 0.04	0.78 ± 0.16	1.06 ± 0.23	1.18 ± 0.18
C22:4*n*-6, Adrenic	0.14 ± 0.05	n.d	n.d	n.d	0.54 ± 0.12	0.23 ± 0.06	0.34 ± 0.09	0.23 ± 0.07	0.34 ± 0.07	0.24 ± 0.06

Not detected (n.d), control (C), high-fat–sucrose diet (HFSD), metformin (MET 20 mg/kg), *Callistemon citrinus* (*C.c* 200 mg/kg), limonene (LIM 0.43 mg/kg), gallic acid (G 0.0069 mg/kg), ellagic acid (E 0.074 mg/kg), *p*-coumaric acid (p-CA 0.00047 mg/kg), mixture (MIX concentration of all the compounds except Met), vehicle (V). All values are expressed as mean ± SEM (*n* = 6; values statistically different (a, b, c, d) among groups (*p* ≤ 0.05) according to Tukey’s test.

**Table 3:** Lipidomic profiling and metabolic index across experimental groups.

Group	SCD-1 Index (C18) Lipogenesis	SCD-1 Index (C16) Lipogenesis	Δ^5^-Desaturase (D5D) (PUFA Pathway)	SFA/MUFA Ratio (Membrane Fluidity)	Inflammatory Index (ARA/DHA)
Control	0.41	0.03	0.00 *	5.11	6.82
HFSD	0.00 *	0.00 *	0.00 *	---	0.00 *
HFSD + V	0.00 *	0.00 *	0.00 *	---	0.00 *
HFSD + MET	3.13	0.05	15.50	0.98	0.00 *
HFSD + MIX	2.49	0.03	18.50	1.13	6.76
HFSD + *C.c*	1.16	0.04	17.06	1.94	6.87
HFSD + LIM	2.62	0.04	17.39	1.14	10.07
HFSD + G	2.76	0.03	29.94	1.12	6.53
HFSD + E	2.71	0.02	22.43	1.14	5.92
HFSD + p-CA	1.77	0.03	18.02	1.39	6.87

* Note: Values marked 0.00 occur because those specific upstream or downstream polyunsaturated fatty acids were not detected (n.d) in the raw dataset. Control (C), high-fat–sucrose diet (HFSD), metformin (MET 20 mg/kg), *Callistemon citrinus* (*C.c* 200 mg/kg), limonene (LIM 0.43 mg/kg), gallic acid (G 0.0069 mg/kg), ellagic acid (E 0.074 mg/kg), *p*-coumaric acid (p-CA 0.00047 mg/kg), mixture (MIX concentration of all the compounds except Met), vehicle (V).

## Data Availability

The data presented in this study are available on request from the corresponding author. The data are not publicly available due to institutional restrictions.
